# Association of the Nonalcoholic Hepatic Steatosis and Its Degrees With the Values of Liver Enzymes and Homeostasis Model Assessment-Insulin Resistance Index

**DOI:** 10.14740/gr685w

**Published:** 2015-10-21

**Authors:** Mario Augusto Ferreira Cruz, Josilda Ferreira Cruz, Larissa Baracho Macena, Demetrius Silva de Santana, Cristiane Costa da Cunha Oliveira, Sonia Oliveira Lima, Alex Vianey Callado Franca

**Affiliations:** a Department of Medicine, Tiradentes University, Aracaju 49032-490, Sergipe, Brazil; b Sergipe Federal University, Sao Cristovao 49100-000, Sergipe, Brazil

**Keywords:** Fatty liver, Ultrasonography, Liver enzymes, Insulin resistance

## Abstract

**Background:**

Nonalcoholic fatty liver disease (NAFLD) is among the most common chronic diseases of the modern world with a wide variety of factors including genetic, environmental and metabolic. The aim of this study was to verify the association between the degrees of hepatic steatosis at the abdominal ultrasound and the values of aminotransferases (aspartate aminotransferase (AST) and alanine transferase (ALT)), gamma glutamyl transpeptidase (GGT) and homeostasis model assessment-insulin resistance (HOMA-IR) index.

**Methods:**

A prospective, descriptive survey study, using a quantitative analytical examination, was conducted from July 2013 to July 2014. In the statistical analysis, values were expressed as median, first and third quartiles. We used the nonparametric Kruskal-Wallis test to compare the medians between the degrees of steatosis, adopted a statistical significance of 5% (P ≤ 0.05) and used the statistical program SPSS 22.0.

**Results:**

We diagnosed 233/800 (29.1%) patients with hepatic steatosis on routine ultrasound, and 65.7% were female. Regarding degrees, 119 had grade 1 (51.0%), 94 grade 2 (40.4%) and 20 grade 3 (8.6%). The median age of the patients with grade 1, 2 or 3 did not vary significantly (P > 0.05). The median body mass index (BMI), although clinically important because of its elevation, did not differ significantly (P > 0.05). ALT levels increased as the degree of hepatic steatosis has advanced as well as the levels of AST, GGT and HOMA-IR. AST values showed a greater association with the severity of fatty liver (P = 0.0001) than the ALT (P = 0.001).

**Conclusions:**

ALT, AST, GGT and HOMA-IR are associated to the degrees of hepatic steatosis on ultrasound and can help in the selection of patients for the liver histological evaluation.

## Introduction

Nonalcoholic fatty liver disease (NAFLD) covers the entire spectrum of fatty liver disease in individuals without significant consumption of alcohol, ranging from simple steatosis to steatohepatitis and liver cirrhosis [1]. NAFLD is present when the deposit of lipids, particularly triglycerides, exceeds 5% of total weight [1, 2].

NAFLD is among the most common chronic diseases of the modern world with a wide variety of factors including genetic, environmental and metabolic, with prevalence by ultrasonography (US) in industrialized countries ranging from 12.2% to 40% [3-9].

An important factor related to the emergence of nonalcoholic steatohepatitis (NASH) is the presence of insulin resistance (IR). This is defined as a reduced biological response to the actions of insulin, causing the fat, muscle and liver tissues to become unable to metabolize glucose and fatty acids, being exacerbated by obesity and the intake of dietary fats [10, 11]. Thus, the association between IR and deposition of triglycerides in the liver, being evaluated by evaluation model index or the homeostasis model assessment-IR (HOMA-IR) is an important instrument [12]. The reference point for the diagnosis of IR by HOMA-IR is controversial, being considered by the Brazilian Society of Diabetes > 3.60 [13].

NAFLD patients are usually asymptomatic, with incidental diagnostic imaging or by changing aminotransferases. Abdominal US is widely used as first-line method for the investigation of NAFLD and others affections of liver, as a simple method that does not use ionizing radiation, less costly and more affordable [14, 15].

When change occurs in aminotransferases, there is usually increased alanine transferase (ALT), while aspartate aminotransferase (AST) levels remain normal or slightly elevated values. However, approximately 80% of patients have normal ALT levels. The use of ALT for diagnosis is controversial to determine severity of NAFLD [2, 16, 17]. The gamma-glutamyl transpeptidase (GGT) may be a biochemical finding early found in this disease, but also not specific, given being influenced by various factors such as hepatobiliary disease, alcoholism and drug use, making it extremely nonspecific [18]. It has been reported that GGT as a marker sensitive to IR, which may be an early biochemical finding of NAFLD [19].

The aim of this study was to investigate the association between the degrees of hepatic steatosis at abdominal ultrasound and amounts of aminotransferases (AST and ALT), GGT and HOMA-IR.

## Material and Methods

A prospective, descriptive survey type, with analytical and quantitative approach, was conducted. We analyzed 800 patients who underwent the examination of US for various clinical conditions, excluding those with alcohol consumption > 40 g/day and previous liver diseases, from July 2013 to July 2014, starting after approval by the Ethics and Research Committee with protocol 010513R. We submitted patients to the US and biochemical analysis of aminotransferases, GGT, fasting glucose and basal insulin. We conducted the examination of US with convex transducer, dynamic (with formation of the continuous and automatic image) of 3.75 MHz frequency, performed by the same examiner (JFC), experienced in the diagnosis of hepatic steatosis image. US of steatosis classified the degrees based on the criteria of Saadeh et al (2002) [20]. The degree 0 is the normal test, the grade 1 is characterized by the display of fine echoes of the hepatic parenchyma with normal viewing of the diaphragm and intrahepatics vessels, grade 2 is characterized by diffuse increase in the fine echoes with impaired view of intrahepatic vessels and diaphragm, and grade 3 is characterized with a significant increase in fine echoes and impaired or absent visualization of intrahepatic vessels. All patients signed the written informed consent (WIC).

The AST and ALT were performed by NADH method (no P-5-P), and the determination of GGT used tubes with separator gel and ABBOTT reagent with the dosage based on the principle that GGT catalyzes the transfer of the gamma glutamyl group from the substrate 3-carboxy-4-nitroanilide glycylglycine receiver for producing 3-carboxy-4-nitroaniline. From the basal insulin to fasting glucose, we used the HOMA-IR index, using the form proposed by Matthews et al (1985) [12] (fasting insulin U/mL × fasting glucose, mmol/L/22.5).

In the statistical analysis, values were expressed as median, first and third quartiles. We used the nonparametric Kruskal-Wallis test to compare the medians between the degrees of steatosis. We adopted a statistical significance of 5% (P ≤ 0.05) and used the statistical program SPSS 22.0.

## Results

**Figure 1:**
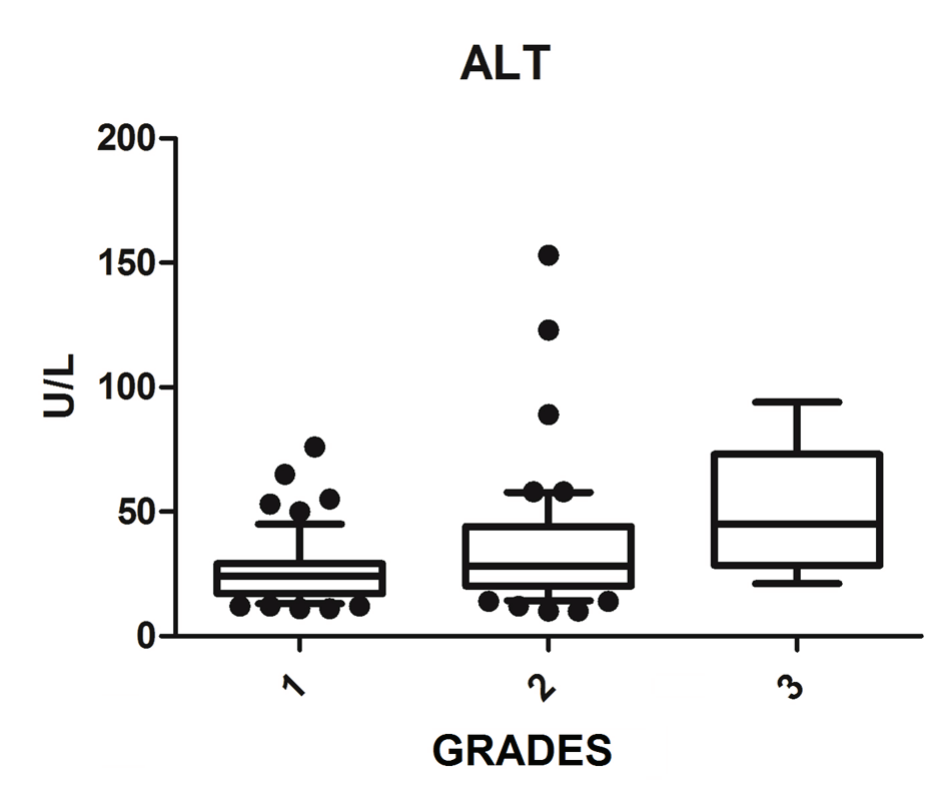
ALT vs. hepatic steatosis. ALT: alanine transferase; P: significance.

**Figure 2:**
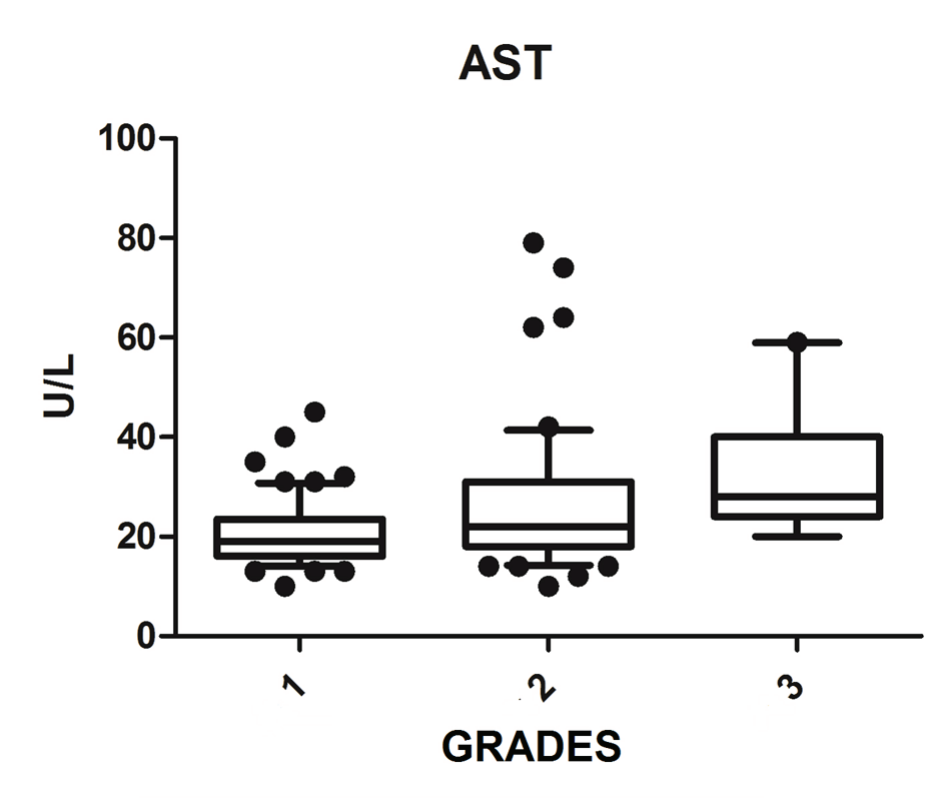
AST vs. hepatic steatosis. AST: aspartate aminotransferase; P: significance.

**Figure 3:**
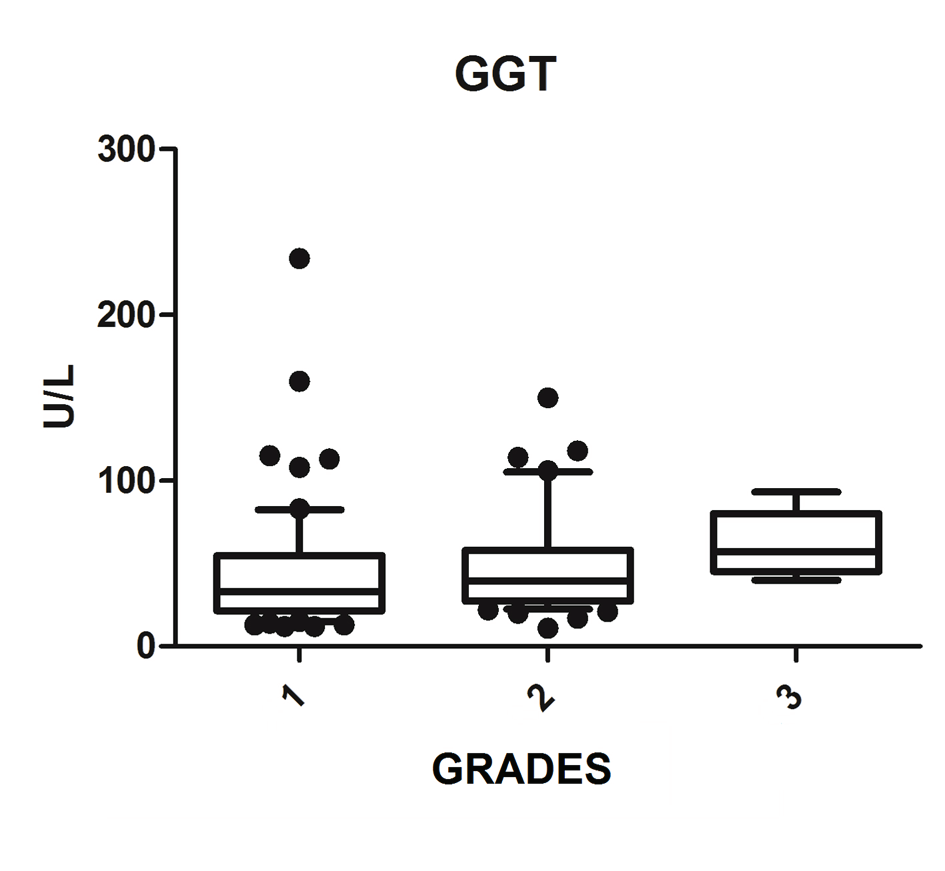
GGT vs. hepatic steatosis. GGT: gamma-glutamyl transpeptidase; P: significance.

**Figure 4:**
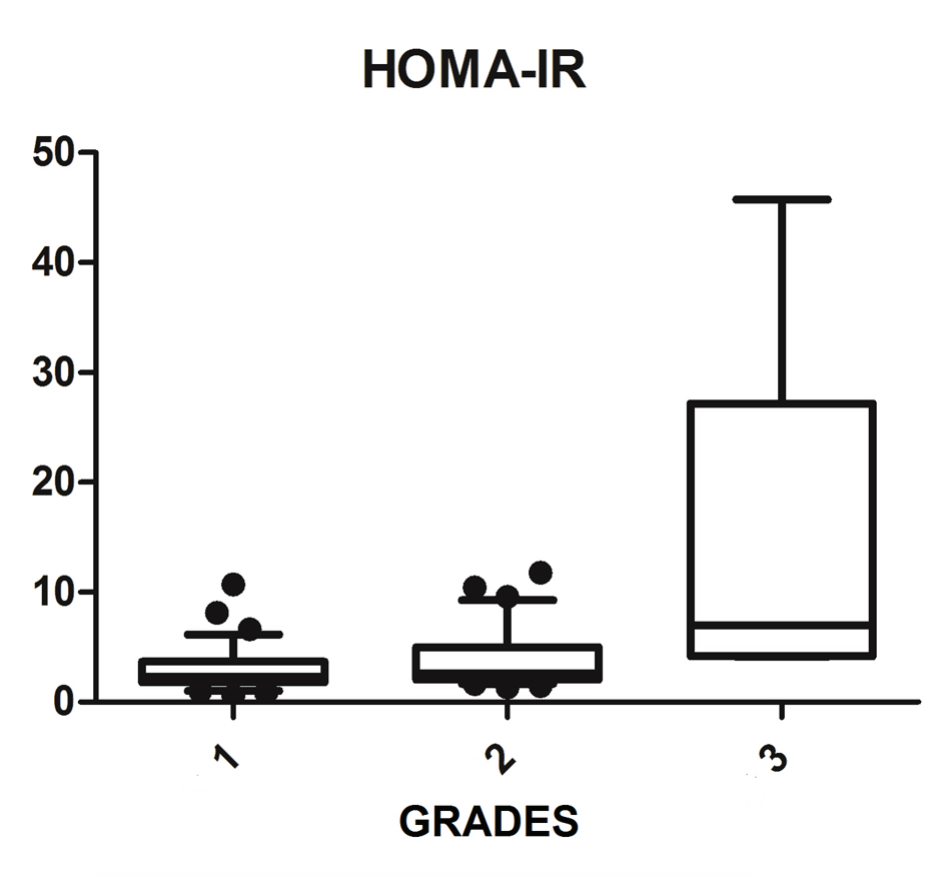
HOMA-IR vs. hepatic steatosis. HOMA-IR: homeostasis model assessment-insulin resistance; P: significance.

We diagnosed 233/800 (29.1%) patients by the US of hepatic steatosis, 153 (65.7%) were female and 80 (34.3%) were male. Regarding degrees, 119 had grade 1 (51.0%), 94 grade 2 (40.4%) and 20 grade 3 (8.6%). The median age of the patients with grade 1 was 44.0 years (first quartile and third quartile were 37.0 and 51.0), grade 2 was 47.5 years (40.0 and 53.0) and grade 3 was 45.5 (36.5 and 54.8), but did not vary significantly (P > 0.05). The median body mass index (BMI), although clinically important because of its elevation, did not differ significantly (P > 0.05). ALT levels increased as the degree of advanced hepatic steatosis (Fig. 1) as well as the levels of AST, GGT and HOMA-IR, shown in Figures 2-4, respectively.

The median AST, ALT, GGT and HOMA-IR index showed statistically significant variation in the degree of hepatic steatosis (P ≤ 0.05), as shown in Table 1. The AST showed a greater association with the severity of steatosis (P = 0.0001) than the ALT (P = 0.001).

**Table 1:** Variables Medians vs. Severity of Hepatic Steatosis

	Nonalcoholic hepatic steatosis						P
	Grade 1		Grade 2		Grade 3		
	Median (quartile)	n	Median (quartile)	n	Median (quartile)	n	
Age (n = 233)	44.0 (37.0 - 51.0)	119	47.5 (40.0 - 53.0)	94	45.5 (36.5 - 54.8)	20	0.107
BMI (n = 125)	29.5 (26.5 - 32.4)	64	30.4 (27.9 - 35.2)	54	33.6 (29.2 - 45.2)	7	0.129
GGT (n = 127)	34.0 (21.5 - 55.5)	65	39.5 (28.5 - 62.0)	56	57.0 (42.2 - 80.2)	6	0.034
AST (n = 135)	19.0 (16.0 - 23.0)	68	23.0 (18.0 - 31.2)	58	28.0 (24.0 - 40.0)	9	0.0001
ALT (n = 136)	24.0 (17.0 - 28.5)	69	29.5 (20.7 - 44.0)	58	45.0 (28.5 - 73.0)	9	0.001
HOMA-IR (n = 75)	2.34 (1.88 - 3.58)	36	2.62 (2.04 - 5.23)	34	6.98 (4.2 - 27.2)	5	0.008

BMI: body mass index; GGT: gamma-glutamyl transpeptidase; AST: aspartate aminotransferase; ALT: alanine transferase; HOMA-IR: homeostasis model assessment-insulin resistance; n: number of patients; P: significance.

## Discussion

The prevalence of alcoholic hepatic steatosis does not vary depending on the region studied and the diagnostic methods used. Eastern countries often have a lower prevalence compared to the West, as the Western lifestyle is, in itself, an important environmental risk factor. The prevalence of NASH by the US is 40% in Germany [3], 27.3% in South Korea [4], 25.8% in Spain [5], 20% in Italy [6], 17.2% in China [7] and 12.2 % in the Philippines [8]. In Brazil, a previous study showed a prevalence of 23% [9]. This study showed that 29.1% of adults surveyed in the city of Aracaju, Sergipe, Brazil, had NAFLD. This disease of the liver has a high frequency in many countries, which was corroborated with the Brazilian present study, one of the most prevalent chronic diseases in the world today.

Currently ultrasound criteria are mainly used to evaluate the severity of hepatic steatosis, for example, the portal vein flow velocity and the hepatic artery resistance index, because current studies show that the portal vein flow velocity and the hepatic artery resistance index decrease with increasing severity [21, 22]..However, it is not a unanimity [23], and it has researched involving the liver and metabolic markers that may assist in this evaluation.

Elevated ALT levels appear to be associated with NAFLD clinically and histologically [24], since those levels reflect the eating habits of the patient, with decreasing levels observed after the consumption of a diet rich in vegetables and low in animal protein [25]. Bi et al (2014) evaluated more than 8,000 people and had higher values of AST and ALT in the group of patients with NAFLD compared to the control group, and found that patients with NAFLD with advanced degrees had significantly increased aminotransferase values, thus, there was strong correlation between the levels of these enzymes and NAFLD [26]. This fact was corroborated by Tomizawa et al (2014) that showed that AST and ALT were significantly higher in patients with NAFLD compared to those without NAFLD (P = 0.0001) [27] and also by this study, which found a significant association of AST and ALT levels with increased ultrasound degrees of hepatic steatosis. The aminotransferase can be used to predict the degree of NAFLD.

However, there is controversy in the literature about the use of aminotransferases in assessing the severity of NAFLD. McPherson et al (2010) and Dyson et al (2014) showed in their studies that the ALT values do not correlate with histological findings and are of little use to determine the severity [2, 17]. The progression to more advanced levels can be noted in several patients with normal ALT, and in addition, the increase in ALT not associated with hepatic steatosis is observed in obese individuals, which impairs their use in clinical practice [28, 29].

The increase in GGT can be found early in NAFLD [18]; however, Bi et al (2014) showed that ALT is a more specific marker than GGT [26]. In the Portuguese population sample, Martins et al (2010) investigated the association of serum GGT to each of the risk factors for metabolic syndrome, being particularly strong with IR and being related to NAFLD [30]. This study found that whenever NAFLD hypothesis is clinically put, GGT values should be obtained, since it is a simple and important marked IR and cardiovascular risk factor [27]. This study found a statistically significant association between increased GGT and degrees of NASH, and may be useful for evaluation and monitoring of patients with NAFLD.

The combination of US of the liver and HOMA-IR is a sensitive and specific method not only to diagnose steatosis, but also in the prediction of severity [31, 32]. Damiani et al (2011) have also shown a strong relationship between IR measured by HOMA-IR and hepatic fat. Fedchuk et al (2014) found a median HOMA-IR 3.3 (2.3 - 5.6) between patients with NAFLD [33]. Moreover, the risk of IR increased 2.33-fold in patients with NAFLD, and coexistence of NAFLD and elevated ALT were significantly associated with increased HOMA-IR amounting to a 4.65 times higher risk of IR. Thus, the study found that the coexistence of NAFLD and elevated ALT are associated with IR and can be useful for early detection of IR [34]. This study found that the HOMA-IR index is statistically associated with the evolution of the degrees of hepatic steatosis with a median of 6.98 (4.2 - 27.2) among patients with liver disease on most ultrasound graduation. Thus, the HOMA-IR index is an important tool that assists in the investigation of NAFLD, and can predict progression to more advanced degrees.

### Conclusion

ALT, AST, GGT and HOMA-IR are related to the degrees of hepatic steatosis on ultrasound, and can help in the selection of patients for liver histological evaluation.
